# Empagliflozin in the Absence of Diabetes: A Systematic Review of Its Anthropometric and Metabolic Effects in Humans and Animals

**DOI:** 10.1155/ije/5558442

**Published:** 2026-08-03

**Authors:** Farima Farsi, Dorsa Ghorban Sarvi, Mohammadmahdi Abbasi, Shirin Hasani-Ranjbar

**Affiliations:** ^1^ Obesity Eating Habits Research Center, Endocrinology and Metabolism Clinical Sciences Institute, Tehran University of Medical Sciences, Tehran, Iran, tums.ac.ir

**Keywords:** anthropometric parameters, empagliflozin, hepatic disorder, metabolic indices, obesity

## Abstract

**Purpose:**

Obesity raises metabolic and cardiovascular risk and represents a major public health challenge. The sodium–glucose cotransport‐2 inhibitor empagliflozin (EMPA) may improve metabolic parameters beyond glycemic control. This systematic review critically evaluated the effects of EMPA on anthropometric and metabolic outcomes in overweight or obese subjects without diabetes and identified key areas for future research.

**Methods:**

This systematic review included studies identified through searches of five databases (Scopus, Web of Science, PubMed, Google Scholar, and the Cochrane Library) from January 2023 to May 2026. Following duplicate removal and PRISMA‐guided screening, 27 studies were included (7 randomized controlled trials and 20 animal studies). Studies were excluded if they involved diabetic populations, lacked appropriate comparator groups, or did not meet predefined eligibility criteria. The Cochrane and SYRCLE tools were used to assess the quality of human and animal evidence, respectively.

**Results:**

Animal studies primarily used EMPA doses of 8–30 mg/kg/day, whereas human trials employed fixed clinical doses of 10–12.5 mg daily. In human investigations, EMPA significantly lowered body weight with notable improvements in fasting glucose. Preclinical studies largely supported these findings and additionally demonstrated improvements in hepatic steatosis, lipid metabolism, and inflammatory markers. Proposed mechanisms included modulation of FGF21 signaling, hepatic PDK4 expression, hypothalamic neuropeptides, NF‐κB activity, mitochondrial function, and gut microbiome composition.

**Conclusion:**

Even in the absence of diabetes, EMPA shows potential for improving anthropometric and metabolic indices. However, clinical evidence remains limited and further human trials are needed to confirm its long‐term safety, efficacy, and underlying molecular mechanisms.

## 1. Introduction

Obesity and overweight are defined by a high level of body fat accumulation that negatively affects health [1]. The global prevalence of overweight and obesity continues to increase among both adults and children [1, 2]. Significant metabolic issues, including fatty liver disease and dyslipidemia, as well as hypertension and insulin resistance, may arise even in individuals without diabetes due to this increasing tendency [3–5], as well as cardiovascular risks [6, 7]. One sodium–glucose cotransporter‐2 (SGLT2) inhibitor approved for the treatment of Type 2 diabetes mellitus (T2DM) is empagliflozin (EMPA). Studies across various patient populations, both as an adjunct to other glucose‐lowering agents and as a standalone treatment, have demonstrated its efficacy. EMPA exerts its effects independently of the insulin pathway and β‐cell activity [8]. By blocking renal glucose reabsorption, EMPA increases urinary glucose excretion [9]. In addition to its glycemic effects, EMPA has attracted considerable scientific interest for its potential benefits in promoting weight loss, improving metabolic control, and offering cardiovascular protection, thereby broadening its application beyond diabetes management [10]. Whether used alone or as an add‐on, EMPA is usually well‐tolerated [11]. The need for efficient weight management strategies is growing among the overweight and obese nondiabetic population [12]; thus, studying the effects of EMPA in this group is clinically relevant. SGLT2 inhibitors have shown promise in earlier research for improving lipid metabolism, insulin sensitivity, and weight loss in this population [13]. However, comprehensive reviews evaluating the therapeutic potential of EMPA in obesity and associated complications are scarce [14]. Despite promising findings, the available evidence remains heterogeneous and limited, especially concerning nondiabetic individuals [15]. A comprehensive assessment of the current data is essential to highlight the impact of EMPA on several anthropometric and metabolic outcomes, including weight change, fat mass, lipid parameters, and liver function in the absence of diabetes [16]. By integrating mechanistic insights and identifying critical gaps, this systematic review sought to assess the emerging evidence on EMPA in nondiabetic populations and to inform future research.

## 2. Methods

### 2.1. Procedure and Registration

All aspects of this systematic review were recorded lucidly and thoroughly in alignment with the requirements established by the Preferred Reporting Items for Systematic Reviews and Meta‐Analyses (PRISMA) framework [17]. The literature search, eligibility assessment, and data extraction were performed by two independent reviewers. When a unanimous decision could not be achieved after thorough discussion, a third reviewer was approached for input. Furthermore, to ensure methodological rigor, review quality of was evaluated using the AMSTAR 2 (A Measurement Tool to Assess Systematic Reviews) checklist. Ethical approval for this systematic review was obtained from the Tehran University of Medical Sciences Ethics Committee (ID: IR.TUMS.EMRI.REC.1403.137). The protocol was registered in the Open Science Framework (OSF) with the registration ID: https://doi.org/10.17605/OSF.IO/M6RFH and was updated during the review process to extend the literature search period in response to peer‐reviewed recommendations. No other substantive deviations from the registered protocol occurred.

### 2.2. Eligibility Criteria

#### 2.2.1. Requirements for Inclusion

The following criteria were used to determine whether a study was eligible for inclusion: (1) randomized controlled trials (RCTs) or (2) preclinical studies; (3) inclusion of participants with overweight or obesity; (4) evaluation of EMPA as the intervention, irrespective of treatment duration; (5) reporting at least one anthropometric outcome and/or at least one metabolic outcome; and (6) publication between January 2023 and May 2026.

#### 2.2.2. Exclusion Criteria

The inclusion of only diabetics or the lack of a separate examination of nondiabetic subjects constituted a criterion for research exclusion. Additional criteria included case reports, case series, observational studies, editorials, commentaries, expert opinions, reviews, qualitative studies, meta‐analyses, conference abstracts, unpublished studies, and the absence of a comparator group. Finally, studies that did not evaluate the effects of EMPA separately were excluded.

### 2.3. Information Sources and Search Strategy

The PubMed, Google Scholar, Scopus, Web of Science, and Cochrane Library databases were systematically searched from January 2023 to May 2026, using the following search terms: (“sodium‐glucose cotransporter‐2 inhibitors” OR “SGLT2 inhibitors” OR “SGLT2i” OR “empagliflozin” OR “EMPA”) AND (“obesity” OR “adiposity” OR “anthropometric” OR “metabolic” OR “liver” OR “hepatic” OR “weight loss” OR “glucose” OR “insulin resistance” OR “HOMA‐IR” OR “lipid profile” OR “triglyceride” OR “cholesterol” OR “inflammation”). Furthermore, related articles for each search result, as well as articles that cited these studies and the references within them, were examined. In the absence of a translation, the search yielded only items authored in English.

### 2.4. Study Selection Process

**FIGURE 1:**
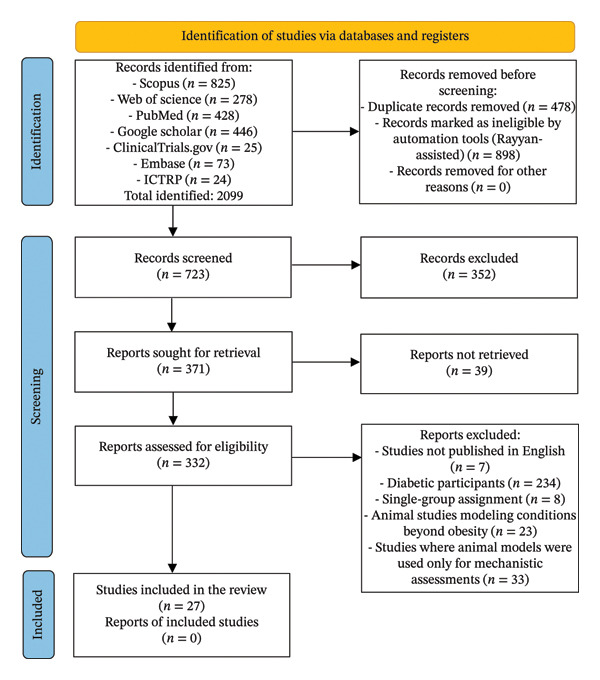
Study selection flow diagram. Flow diagram constructed by the authors according to PRISMA 2020 guidelines [18].

The selection process is illustrated in Figure 1. Duplicate papers and those deemed ineligible by Rayyan were excluded from the first selection. Rayyan identified ineligible studies based on two key criteria: study design and outcome measurement. Additionally, studies that failed to assess the primary outcomes were excluded. In addition to database searches, ClinicalTrials.gov was reviewed to identify ongoing or unpublished trials. The second stage of the selection process involved title and abstract screening. Finally, the remaining eligible articles underwent full‐text screening. To ensure accuracy and consistency, two independent researchers reviewed the screening results and resolved any discrepancies through discussion.

### 2.5. Data Extraction Process

Data on study characteristics (first author, publication year, and country), sample size, intervention duration and follow‐up, population characteristics, EMPA dosages, and study outcomes were extracted by two independent authors using a predefined data extraction template. Additionally, information regarding the underlying mechanisms of EMPA’s effects was collected. All disputes were settled via dialog until consensus was achieved.

### 2.6. Risk‐of‐Bias (RoB) Assessment

Bias in clinical studies was assessed using the Cochrane RoB 2 [19] and the SYRCLE RoB Tool for animal studies [20]. The Cochrane assessment tool covers five domains of bias: (1) bias stemming from the randomization process, (2) deviations from the planned interventions, (3) missing outcome data, (4) outcome measurement, and (5) selection of reported outcomes. The domains assessed by the SYRCLE RoB Tool include (1) generating sequence, (2) defining baseline features, (3) concealing allocation, (4) random housing of animals, (5) blinding both caregivers and investigators, (6) blinding of outcome assessment, (7) conducting random outcome assessments, (8) incomplete outcome data, and (9) exclusively outcome reporting.

## 3. Results

### 3.1. Study Selection

Figure 1 presents the PRISMA flow diagram illustrating the study selection process. A total of 2099 records were initially retrieved from multiple databases and registers: Scopus (*n* = 825), Web of Science (*n* = 278), PubMed (*n* = 428), Google Scholar (*n* = 446), ClinicalTrials.gov (*n* = 25), Embase (*n* = 73), and ICTRP (*n* = 24). After the removal of 478 duplicate records and 898 ineligible studies flagged by automation tools, 723 records underwent title and abstract screening.

Following screening, 352 records were excluded, leaving 371 reports for full‐text retrieval. Of these, 39 reports could not be accessed, and 332 were assessed for eligibility. Ultimately, 305 reports were excluded for the following reasons:

• Not published in English (*n* = 7)
• Studies conducted exclusively in populations with diabetes (*n* = 234)
• Studies lacking a comparator group (*n* = 8)
• Studies employing obesity models other than high‐fat diet‐induced obesity or fatty liver disease (*n* = 23)
• Studies focusing solely on molecular mechanisms of EMPA without clinical assessments (*n* = 33)

Consequently, the final systematic review included 27 studies.

**FIGURE 2:**
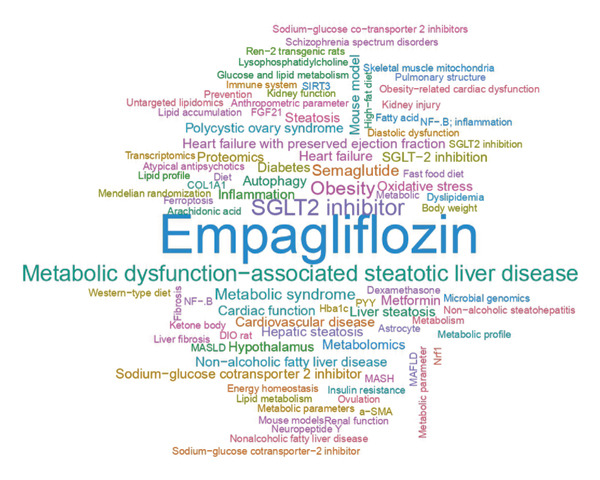
Keywords of the studies included in this research.

Figure 2 presents a word cloud, which was generated from author keywords and key terms extracted from the included studies. The size of each word reflects its frequency within the reviewed literature. As expected, “Empagliflozin” emerged as the dominant keyword, followed by terms related to metabolic dysfunction–associated steatotic liver disease, SGLT2 inhibition, obesity, metabolic syndrome, and semaglutide. Other commonly reported keywords included hepatic steatosis, liver steatosis, inflammation, cardiac function, heart failure, cardiovascular disease, metabolomics, and autophagy. Collectively, these terms highlight the broad focus of the included studies on the anthropometric, metabolic, hepatic, cardiovascular, and mechanistic effects of EMPA in nondiabetic settings.

### 3.2. Characteristics of Included Studies

Tables 1 and 2 summarize the baseline characteristics of the included RCTs and animal studies. The RCTs investigated the effects of EMPA in diverse nondiabetic populations, including individuals with polycystic ovary syndrome (PCOS), metabolic dysfunction–associated steatotic liver disease (MASLD), heart failure with preserved ejection fraction (HFpEF), schizophrenia spectrum disorders receiving atypical antipsychotics, and older adults with overweight or obesity at increased risk of heart failure. The study populations varied in terms of age, sex distribution, and comorbidities. Sample sizes ranged from 52 to 5988 participants. EMPA was administered at daily doses of 10–12.5 mg as monotherapy or in combination with lifestyle interventions and concomitant therapies. Treatment duration ranged from 16 weeks to more than 2 years. Key exclusion criteria differed across trials but commonly involved comorbidities that could confound metabolic or cardiovascular outcomes, including diabetes, significant cardiovascular conditions, severe renal impairment, active liver disease unrelated to metabolic dysfunction, and pregnancy or lactation.

**TABLE 1:** Baseline characteristics of RCTs investigating the effects of empagliflozin on anthropometric and metabolic indices.

Author, year	Publication	*n*	Intervention/comparator	Duration
El Gohary et al., 2023 [21]	Women with PCOS, aged 18–35 years, BMI ≥ 27 kg/m^2^	150 (EMPA group: 75; control group: 75)	EMPA 12.5 mg + metformin 500 mg OD + clomiphene citrate 50 mg BID (days 2–6) vs. metformin 500 mg OD + clomiphene citrate 50 mg BID (days 2–6)	3 months
Cheung et al., 2024 [22]	Adults with MASLD without diabetes, predominantly with overweight/obesity (≥ 18 years; 55.1% male; median BMI ≈ 27.4 kg/m^2^)	98 (EMPA: 49; placebo: 49)	EMPA 10 mg once daily vs. placebo	52 weeks
Böhm et al., 2023 [23]	Older adults with HFpEF (mean age 71–72 years; 45% female; LVEF > 40%; median NT‐proBNP 972 pg/mL)	5988 (EMPA: 2997; placebo: 2991)	EMPA 10 mg once daily vs. placebo	Median 26.2 months
Sharif et al., 2025 [24]	Women with PCOS and overweight/obesity (mean age 24.9 years; BMI > 25 kg/m^2^) with insulin resistance/prediabetes	80 (EMPA: 41; metformin: 39)	Lifestyle modification + EMPA 10 mg once daily vs. lifestyle modification + metformin 500 mg twice daily	24 weeks
Wong et al., 2026 [25]	Adults with schizophrenia spectrum disorders receiving atypical antipsychotics (mean age ∼42 years; 50% female; BMI ≥ 23 kg/m^2^)	52 (EMPA: 26; placebo: 26)	EMPA 10 mg once daily + standardized lifestyle intervention vs. placebo + standardized lifestyle intervention	16 weeks
Taha et al., 2025 [26]	Adults with MAFLD without diabetes (median age 42 years; ∼64%–70% male; BMI 29.4–31.8 kg/m^2^)	55 (EMPA + lifestyle: 33; lifestyle only: 22)	Lifestyle advice + EMPA 10 mg once daily vs. lifestyle advice only	6 months

*Related Articles*				
Larsen et al., 2026 [27]	Older adults with overweight and high risk of heart failure (median age 68 years; 67% male; BMI > 28 kg/m^2^, median 31.8 kg/m^2^)	191 randomized (EMPA: 96; placebo: 95); 165 completed MRI analysis (EMPA: 83; placebo: 82)	EMPA 10 mg once daily vs. placebo	180 days

*Note:* NT‐proBNP = N‐terminal Pro‐B‐type natriuretic peptide, HFpEF = heart failure with preserved ejection fraction, HbA1C = glycated hemoglobin, HBs = hepatitis B surface. Placebo: manufactured to match the appearance of empagliflozin.

Abbreviations: BMI = body mass index, CAP = controlled attenuation parameter, eGFR = estimated glomerular filtration rate, FBS = fasting blood glucose, HCV = hepatitis C virus, MRI‐PDFF = magnetic resonance imaging proton density fat fraction, OGTT = oral glucose tolerance test.

**TABLE 2:** Baseline characteristics of animal studies investigating the effects of empagliflozin on anthropometric and metabolic indices.

Author, year	Species/strain	Disease model	*n*	Intervention/comparator	Duration
Nguyen et al., 2024 [28]	Male C57BL/6J mice	Diet‐induced obesity (45% or 60% HFD)	48 (EMPA: 24; control: 24)	EMPA (∼0.3–0.35 mg/day in chow) vs. HFD control	16 weeks
Kloock et al., 2024 [29]	Male Wistar rats	Diet‐induced obesity with early MASLD (45% HFD)	34 (EMPA: 5; HFD control: 6)	EMPA 10 mg/kg/day in drinking water vs. HFD control	8 weeks
Kim et al., 2024 [30]	Male C57BL/6N mice	Diet‐induced obesity (16‐week HFD)	28 (oral EMPA: 5; HFD control: 5; i.c.v. EMPA: 11; vehicle: 8)	EMPA 10 mg/kg/day orally or 50 nM/2 μL i.c.v. vs. HFD or vehicle control	3 weeks
Luo et al., 2024 [31]	Male C57BL/6J mice	Diet‐induced obesity with obesity‐related cardiac dysfunction (HFD)	24 (EMPA: 8; HFD control: 8; normal control: 8)	EMPA 10 mg/kg/day orally vs. HFD control	8 weeks
Yue et al., 2024 [32]	Male C57BL/6 mice	Diet‐induced obesity with aortic injury (HFD)	24 (EMPA: 8; HFD control: 8; normal control: 8)	EMPA 10 mg/kg/day via oral gavage vs. HFD control	12 weeks
Radlinger et al., 2023 [33]	Male C57BL/6 mice	Diet‐induced obesity, insulin resistance, and hepatic steatosis (Western diet)	140^∗^	EMPA 30 mg/kg/day via diet vs. Western diet and control diet groups without EMPA	10 weeks
Kim et al., 2024 [34]	Male C57BL/6N mice	Diet‐induced obesity with hypothalamic inflammation (HFD)	20 (HFD + EMPA: 5; HFD: 5; NFD + EMPA: 5; NFD: 5)	EMPA 10 mg/kg/day via oral gavage vs. NFD and HFD controls without EMPA	3 weeks
Shi et al., 2023 [35]	Male C57BL/6J mice	Diet‐induced obesity with impaired intestinal homeostasis (HFD)	24 (EMPA: 8; HFD: 8; control: 8)	EMPA 10 mg/kg/day intragastrically vs. HFD and control groups	8 weeks
Heo et al., 2025 [36]	Male C57BL/6J mice	Diet‐induced MASLD with hepatic steatosis and oxidative stress (HFD)	40 (EMPA: 16; control: 24)	EMPA 10 mg/kg orally every other day vs. HFD control (AAV‐shGFP)	6 weeks
Hupa‐Breier et al., 2025 [37]	Female TH mice	Diet‐induced MASLD with severe obesity and steatohepatitis (HF‐HC diet)	15 (EMPA: 8; control: 7)	EMPA 225 mg/kg diet orally (mixed in chow) vs. HF‐HC diet control	4 weeks
Makri et al., 2025 [38]	Male and female C57BL/6J mice	Fast‐food diet–induced obesity and hepatic steatosis (FFD)	24 (EMPA: 8; FFD: 8; control: 8)	EMPA 10 mg/kg/day incorporated into FFD pellets vs. FFD and control diet groups	6 months
Niu et al., 2025 [39]	Male C57BL/6J mice	Diet‐induced MASLD (60% HFD)	32 (EMPA: 8; semaglutide: 8; HFD: 8; control: 8)	EMPA 10 mg/kg/day via oral gavage vs. HFD and control groups	12 weeks
Fu et al., 2025 [40]	Male leptin‐deficient ob/ob mice (with C57BL/6J mice as normal controls)	Diet‐induced NAFLD with liver inflammation and fibrosis (GAN diet)	24 (EMPA: 6; semaglutide: 6; model: 6; normal control: 6)	EMPA 8 mg/kg/day via oral gavage vs. GAN diet model control	5 weeks

*Related Articles*					
Hojná et al., 2024 [41]	Male spontaneously hypertensive rats (SHR)	High‐fat diet‐induced hepatic steatosis and mild heart failure	27 (EMPA: 9; HF control: 10; normal control: 8)	EMPA 10 mg/kg/day in drinking water vs. high‐fat diet control	2 months
Yang et al., 2024 [42]	Male C57BL/6JC mice	Diet‐induced obesity with pulmonary structural alterations (HFD)	28 (EMPA: 7; semaglutide: 7; HFD: 7; control: 7)	EMPA 10 mg/kg/day via oral gavage vs. HFD and control groups	12 weeks
Miklankova et al., 2024 [43]	Male Wistar and hereditary hypertriglyceridemic (HHTg) rats	Prediabetes with severe dyslipidemia	32 (Wistar: 8; Wistar + EMPA: 8; HHTg: 8; HHTg + EMPA: 8)	EMPA 10 mg/kg/day via diet vs. untreated Wistar and HHTg controls	8 weeks
Zambrano‐Vásquez et al., 2025 [44]	Male Wistar rats	Diet‐induced metabolic syndrome and MASLD (Western diet)	30 (EMPA + metformin: 6; EMPA: 6; metformin: 6; MASLD control: 6; healthy control: 6)	EMPA + metformin (12.5/850 mg/kg/day) via oral gavage vs. untreated MASLD control	30 days
Berger et al., 2025 [45]	Male C57BL/6NJ mice	HFpEF induced by diet‐induced obesity and renin‐mediated hypertension (two‐hit model)	10–15 per group for echocardiographic analyses; EMPA cohort: 12–13 per group^∗^	EMPA (50–500 mg/kg diet) administered continuously in HFD vs. untreated HFpEF model (HFD + AAV‐Renin + vehicle)	14 weeks
Hojná et al., 2026 [46]	Male Ren‐2 transgenic hypertensive rats	Angiotensin II‐dependent hypertension (nondiabetic)	28 (ACEi: 8; ACEi + EMPA: 9; triple therapy: 5; triple therapy + EMPA: 6)	EMPA 10 mg/kg/day orally combined with ACEi (trandolapril) or triple antihypertensive therapy vs. corresponding antihypertensive therapy alone	8 weeks
Alharbi et al., 2026 [47]	Male Wistar rats	Dexamethasone‐induced NASH	16 (control: 4; dexamethasone: 4; EMPA 10 mg/kg + dexamethasone: 4; EMPA 30 mg/kg + dexamethasone: 4)	EMPA 10 or 30 mg/kg/day orally + dexamethasone vs. dexamethasone‐only control	13 days

*Note:* EMPA = empagliflozin, Met = metformin, AgRP = agouti‐related peptide, POMC = pro‐opiomelanocortin, i.c.v. = intracerebroventricular, shGFP = short‐hairpin RNA targeting green fluorescent protein, shNrf1 = short‐hairpin RNA targeting nuclear respiratory factor 1, MASLD = metabolic dysfunction–associated steatotic liver disease, i.v. = intravenous, IP = intraperitoneal, Nrf1 = nuclear respiratory factor 1, MASLD = metabolic dysfunction–associated steatotic liver disease, HF‐HC = high‐fat high‐carbohydrate diet, TH = TALLYHO/JngJ mice, HEC = hydroxyethyl cellulose, ACEi = angiotensin‐converting enzyme inhibitor, GAN = Gubra‐Amylin nonalcoholic steatohepatitis diet.Abbreviations: AAV = adeno‐associated virus, FFA = free fatty acids, FFD = fast‐food diet, HFD = high‐fat diet, NAFLD = nonalcoholic fatty liver disease, NFD = normal‐fat diet, SPF = specific pathogen‐free.

^∗^Exact allocation per group not reported.

Most of the animal studies utilized C57BL/6‐derived mouse models, while others employed female TH mice, leptin‐deficient ob/ob mice, Wistar rats, spontaneously hypertensive rats, hereditary hypertriglyceridemic rats, and Ren‐2 transgenic hypertensive rats (Table 2). The experimental models primarily focused on obesity, MASLD, nonalcoholic steatohepatitis (NASH), metabolic syndrome, hypertension, and HFpEF, induced by dietary, pharmacological, or genetic approaches. EMPA was given orally through drinking water, dietary admixture, or oral gavage at doses ranging from 8 to 30 mg/kg/day either as monotherapy or in combination with metformin, semaglutide, antihypertensive therapy, or genetic interventions. Treatment durations varied considerably across studies, ranging from 13 days to 25 weeks.

### 3.3. Study Outcomes

Tables 3 and 4 summarize the anthropometric, metabolic, lipid, hepatic, and other outcomes reported in the included human and animal studies, respectively. These tables provide a structured overview of the measured effects of EMPA across clinical and preclinical settings. A detailed interpretation and discussion of these findings are presented in the Discussion section.

**TABLE 3:** Summary of empagliflozin‐related outcomes in included RCTs.

Study	Weight/BMI	Fat mass	Glucose/insulin	Lipids	Liver	Others
El Gohary et al. [21]	↓ BMI (30.81 ± 2.22 vs. 34.19 ± 7.86 kg/m^2^, *p* = 0.001) ↓ hip circumference (Δ significant, *p* = 0.004) ↓ waist circumference (Δ marginal, *p* = 0.05) ↔ WHR	Not reported	↓ FBS (75.82 ± 14.9 vs. 80.18 ± 10.5 mg/dL, *p* = 0.04) ↓ fasting insulin (17.81 ± 8.88 vs. 15.1 ± 7.57 mIU/mL, *p* = 0.002) ↔ HOMA‐IR	Not reported	Not reported	—

Cheung et al. [22]	↓ Body weight (Δ −2.7 kg vs. −0.2 kg, *p* < 0.001) ↓ BMI (Δ −1.0 vs. −0.1 kg/m^2^, *p* < 0.001, significant from Week 6) ↓ waist circumference (Δ −2.0 vs. 0.0 cm, *p* = 0.011, significant from Week 12)	Not reported	↓ FBS (Δ −0.3 vs. 0 mmol/L, *p* < 0.001, significant from Week 12) ↔ HbA1c	↔ Total cholesterol ↔ LDL ↔ HDL ↔ triglycerides	↓ MRI‐PDFF (Δ −2.49% vs. −1.43%, *p* = 0.025) ↔ resolution of hepatic steatosis ↔ ALT reduction ≥ 17 U/L ↔ MRI‐PDFF decline ≥ 30% ↔ composite outcome (ALT reduction + MRI‐PDFF decline ≥ 30%) ↓ ALP (−2.0 vs. +1.0 U/L, *p* = 0.034) ↓ ALT (−5.0 vs. 0 U/L, *p* = 0.054) ↓ AST (−3.0 vs. 0 U/L, *p* = 0.069) ↔ GGT ↓ serum ferritin (−126 vs. −22 pmol/L, *p* = 0.035, significant from Week 12)	↔ SBP and DBP ↔ adverse event incidence ↔ serious adverse events

Böhm et al. [23]	Not reported	Not reported	Not reported	Not reported	↔ ALP ↑ bilirubin at baseline associated with poor outcomes ↔ AST and ALT	↓ Albumin at baseline associated with poor outcomes

Sharif et al. [24]	↓ Weight (Δ −6.7 ± 1.6 kg) ↓ BMI (Δ −2.4 ± 0.7 kg/m^2^) Greater than metformin	Not reported	FBS ↓ (Δ −26.4 ± 3.6 mg/dL); HbA1c ↓ (Δ −0.9 ± 0.2%)	↓ Total cholesterol (Δ −34.9 ± 3.7 mg/dL) ↓ LDL (Δ −31.1 ± 4.9 mg/dL) ↓ triglycerides (Δ −72.8 ± 7.0 mg/dL) ↑ HDL (Δ +25.2 ± 2.0 mg/dL) Greater than metformin	Not reported	One urinary tract infection reported

Wong et al. [25]	↓ Body weight (Δ −1.53 kg; 95% CI, −2.60 to −0.47, *p* = 0.006) ↓ BMI (Δ −0.53 kg/m^2^; 95% CI, −0.91 to −0.15, *p* = 0.007) ↔ waist circumference	Not reported	↓ FBS (Δ −0.40 mmol/L, *p* = 0.002) ↓ HbA1c (Δ −0.11%, *p* = 0.019)	↔ LDL ↔ HDL ↔ TG	Not reported	↔ SBP and DBP ↔ caloric intake and physical activity levels ↔ adverse events and dropout rates

Taha et al. [26]	↓ Weight (Δ 2.5 kg vs. 1 kg, *p* = 0.022) ↓ BMI (29.7 vs. 30.4 kg/m^2^, *p* = 0.069) ↓ waist circumference (1.01 vs. 1.08 m, *p* = 0.01; Δ 0.02 vs. 0.01 m, *p* = 0.011) ↓ WHR (0.60 vs. 0.63, *p* = 0.032; Δ 0.01 vs. 0.01, *p* = 0.008)	Not reported	↔ HbA1c	↔ Triglycerides ↔ total cholesterol ↔ LDL ↔ HDL	↓ CAP/hepatic steatosis (Δ −38 vs. −8 dB/m, *p* = 0.001; follow‐up 270 vs. 286 dB/m, *p* = 0.004) ↓ liver fibrosis (Δ 1.05 vs. 0.2 kPa, *p* = 0.001) ↔ ALT (follow‐up) ↓ ALT (change) (Δ −13 vs. −6 U/L, *p* = 0.001)	↓ SBP (130 vs. 130 mmHg, *p* = 0.034) ↓ DBP (80 vs. 80 mmHg, *p* = 0.001) Side effects: 21% (6 dizziness; one urinary tract infection); no discontinuation

*Related articles*						
Larsen et al. [27]	↓ Weight (−1.8 kg, *p* = 0.0003)↓ BMI (−0.6 kg/m^2^, *p* = 0.001)	Not reported	↔ HbA1c↔ New‐onset diabetes	Not reported	Not reported	↔ SBP, DBP, MAP, PP ↑ Genital infections (10.8% vs. 0%, *p* < 0.05)↔ NT‐proBNP, eGFR, hemoglobin Cardiac MRI outcomes:↓ LVMI (−1.9 g/m^2^, *p* = 0.01)↔ LVEDVI↔ LVESVI↔ LVEF

*Note:* Placebo: manufactured to match the appearance of empagliflozin. Symbols: ↓ indicates a significant decrease, ↑ indicates a significant increase, ↔ indicates no significant change, Δ denotes change, *p* (*p*‐value), and > indicates a greater effect. WHR = waist‐to‐hip ratio, HOMA‐IR = homeostatic model assessment for insulin resistance, ALT = alanine aminotransferase, AST = aspartate aminotransferase, ALP = alkaline phosphatase, NT‐proBNP = N‐terminal pro‐B‐type natriuretic peptide, HbA1c = glycated hemoglobin.

Abbreviations: BMI = body mass index, CAP = controlled attenuation parameter, DBP = diastolic blood pressure, FBS = fasting blood sugar, GGT = Gamma‐glutamyl transferase, LVEDVI = left ventricular end‐diastolic volume index, LVEF = left ventricular ejection fraction, LVESVI = left ventricular end‐systolic volume index, LVMI = left ventricular mass index, MRI‐PDFF = magnetic resonance imaging proton density fat fraction, PP = pulse pressure, SBP = systolic blood pressure.

**TABLE 4:** Summary of empagliflozin‐related outcomes in included animal studies.

Study	Weight/BMI	Fat mass	Glucose/insulin	Lipids	Liver	Others
Nguyen et al. [28]	↔ Body weight	↓ Dio2 gene expression in white adipose tissue (30% decrease, ^∗^)	↑ Glycosuria (increased glucose excretion: 350–500 mg/day in 60% HFD + EMPA group, equivalent to 1.4–1.9 kcal/day lost, ^∗^)	Not reported	Not reported	↓ FGF21 serum levels (NS) ↔ activity in open‐field test

Kloock et al. [29]	↔ Body weight	Not reported	↓ HOMA index (18% decrease vs. baseline, ^∗^)	↑ NEFA (NS)	↔ MASLD score	↓ TNF‐α transcription (^∗^) ↔ IL‐1β ↔ FGF21

Kim et al. [30]	↓ Body weight 24h after intracerebroventricular (i.c.v.) EMPA administration (^∗^) ↓ weight gain (^∗∗∗^ at Week 2, ^∗∗∗∗^ at Week 3) ↑ body weight in HFD vs. NFD groups, with significant divergence from Week 4 (*p* < 0.05)	Not reported	Not reported	Not reported	Not reported	↑ cumulative dietary intake at 3h after i.c.v. EMPA (^∗^) ↑ dietary intake trend up to 24h after i.c.v. EMPA (NS) ↑ dietary intake during Weeks 1–2 with oral EMPA in HFD group (NS) ↓ dietary intake during Week 3 with oral EMPA in HFD group (NS)

Luo et al.,[31]	↓ Body weight gain (^∗^)	↓ Fat mass (^∗^)	↑ Insulin tolerance (^∗^) ↑ Glucose tolerance (^∗^)	↓ TG (^∗^)	Not reported	↔ FFAs

Yue et al. [32]	↓ Body weight by 12 weeks (^∗∗^)	Not reported	↓ Postprandial glucose (^∗^) ↔ Fasting insulin	↓ Total cholesterol (^∗∗∗^) ↔ TG ↓ LDL‐C (^∗∗∗^)	Not reported	—

Radlinger et al. [33]	↔ Body weight (^∗∗∗^ WD vs. WDE, ^∗∗^ CD vs. CDE)	↓ Fat mass in (^∗∗^)	↓ Blood glucose (at Week 10, ^∗^) ↓ Insulin levels (^∗^) ↑ Glucose infusion rate (GIR), indicating improved insulin sensitivity (^∗^) ↔ Insulin receptor expression	Not reported	↓ Hepatic steatosis (^∗^) ↓ Intrahepatic lipid accumulation (consistent with histology)	↔ glucagon levels ↔ ketone bodies ↔ daily food intake ↑ water intake in EMPA groups (^∗^) ↑ *p*‐Akt/tAkt signaling (^∗∗^) Positively affecting size and morphology of mitochondria in skeletal muscle in both groups

Kim et al. [34]	↔ Body weight after 3 days of HFD ↓ Body weight gain at 16 weeks (^∗^)	Not reported	↓ Blood glucose levels after 16 weeks (^∗^)	Not reported	Not reported	↔ Hypothalamic IL‐1β expression after 3 days ↔ Hypothalamic TNF‐α expression after 3 days ↓ Hypothalamic IL‐1β expression after 3 and 16 weeks (^∗^) ↓ Hypothalamic TNF‐α expression after 3 and 16 weeks (^∗^)

Shi et al. [35]	↓ Body weight (^∗^)	↓ Fat mass (^∗^)	↓ HOMA index (^∗^) ↓ Fasting insulin (^∗^) ↓ Blood glucose following insulin injection (^∗^) ↓ Blood glucose following glucose administration (^∗^) ↔ Fasting blood glucose	↔ Serum TG	Not reported	↓ FFAs (^∗^)

Heo et al. [36]	↓ Body weight in HFD/GFP + EMPA vs. HFD/GFP (^∗∗^) No effect in Nrf1‐knockdown mice	↓ Hepatic fat accumulation (^∗∗∗^)	↑ Glucose and insulin tolerance (^∗^) Benefits lost with Nrf1 knockdown	↓ Hepatic TG ↓ Lipogenesis‐related genes	↓ Steatosis, liver weight, ALT/AST ↓ Oxidative and ER stress	↓ Steatosis, liver weight, ALT/AST ↓ Oxidative and ER stress

Hupa‐Breier et al. [37]	↔ Body weight	↔ Adipose tissue mass (WAT, VAT, BAT)	↓ Fasting blood glucose (^∗^) ↔ HOMA‐IR	↓ Intrahepatic TG (^∗^)	↓ Liver weight (^∗^) ↓ NAS steatosis score (3 vs. 1) ↓ Overall NAS score (5 vs. 3; ^∗^) ↓ Hepatic fibrosis area (^∗^) ↔ serum AST/ALT levels	↓ Absolute numbers of intrahepatic B cells, CD4^+^ T cells, CD8^+^ T cells, and Tregs (^∗^ to ^∗∗∗^) ↓ Proinflammatory gene expression: TNF, CCL2, NOS2 (^∗^ to ^∗∗^) ↓ Pro‐fibrotic gene expression: Col1a1, TIMP1 (^∗^) ↓ FGF21 expression (^∗^) ↑ Antioxidant enzyme expression: CAT, SOD (^∗∗^)

Makri et al. [38]	↔ Body weight gain	Not reported	Not reported	Altered serum and hepatic lipidomic profile (^∗∗∗^) including ↓ LPC and LPE species and ↑ PC and PI species Common changes observed in serum and liver	↔ Steatosis, lobular inflammation, NAS, and fibrosis vs. FFD ↑ Steatosis and lobular inflammation vs. CD ↑ NAS vs. CD	Sex‐specific lipidomic differences (^∗∗∗^) Dietary effects on lipidome exceeded EMPA effects

Niu et al. [39]	↓ Body weight (^∗∗^) Progressive reduction observed during 12‐week intervention	Not reported	↑ Glucose tolerance ↓ Insulin resistance (^∗∗^) ↔ Fasting blood glucose	↓ TC, ↓ LDL‐C, ↑ HDL‐C (all ^∗∗^) ↓ TG vs. HFD but to a limited extent	↓ Hepatic steatosis, lipid droplet accumulation, and hydropic degeneration ↓ ALT and AST (^∗∗^) ↓ Mitochondrial swelling and ↑ mitochondrial microautophagy ↓ Multiple hepatic LPC species (^∗^)	↓ Proinflammatory markers (TNF‐α, IL‐6, IL‐1β) and ↓ oxidative stress (↓ MDA, ↑ SOD) (all ^∗^) ↔ Uric acid levels Sema vs. EMPA: Sema produced greater body weight loss and TG reduction (^∗∗^) Sema significantly reduced serum UA, while EMPA did not No significant differences between cohorts for glucose tolerance, insulin, TC, LDL‐C, HDL‐C, liver enzymes, inflammation, or oxidative stress Sema showed slightly superior histopathological improvement except for lymphocytic infiltration Both drugs shared similar LPC‐lowering patterns

Fu et al. [40]	↓ Body weight and percent body weight change in EMPA group vs. model group (^∗^) Effects comparable to semaglutide group	Not reported	Not reported	↓ Hepatic TG levels (^∗^) ↔ Hepatic TC levels (downward trend)	↓ Hepatic steatosis and NAS score (^∗^) ↓ ALT and AST levels (^∗^) ↓ Collagen deposition/fibrotic area (^∗^) ↓ α‐SMA and COL1A1 expression	↓ Food intake (partial reduction) ↓ TNF‐α, IL‐6, and IL‐1β expression (^∗^) ↔ NF‐κB expression ↓ FASN expression (^∗^) ↔ SREBP‐1c expression (downward trend) ↓ MDA levels (^∗^) ↑ GSH levels (^∗^)

*Related articles*						
Hojná et al. [41]	↓ Body weight gain (final Δ = 17 ± 3 g, ^∗∗^ from Week 5) ↓ Body weight gain percentage (^∗∗∗^)	↔ Perirenal fat mass ↔ Epididymal fat mass	↔ Fasting blood glucose levels (5.7 ± 0.1 vs. 5.3 ± 0.1 mmol/L) ↑ Glucose tolerance (↓ OGTT AUC in EMPA‐treated group, ^∗^) ↔ Serum insulin levels (0.616 ± 0.066 vs. 0.505 ± 0.050) ↔ Muscle insulin resistance ↔ Adipose insulin resistance	↓ Serum TG (0.72 ± 0.03 vs. 0.59 ± 0.02, ^∗^) ↑ HDL‐C (0.50 ± 0.01 vs. 0.56 ± 0.01, ^∗^)	↓ Hepatic TG deposition (^∗^) ↔ ALT levels ↔ AST levels ↓ Hepatic steatosis score (0.9 ± 0.1 vs. 0.67 ± 0.17, ^∗∗^) ↓ NAFLD score (0.9 ± 0.1 vs. 0.67 ± 0.17, ^∗∗^) ↔ Hepatic inflammation and ballooning	↓ Food intake after HF diet transition (40% decrease, ^∗∗∗^)

Yang et al. [42]	↓ Body weight (^∗∗∗∗^) ↓ Body weight reduction greater with semaglutide vs. EMPA from Week 18 (^∗^) ↔ Body weight with semaglutide vs. NCD from Week 17	Not reported	↑ Glucose tolerance impairment in HFD vs. NCD, semaglutide, and EMPA groups at 15 min (^∗^) ↑ glucose tolerance impairment in HFD vs. NCD, semaglutide, and EMPA groups at 30–120 min (^∗∗∗∗^) ↔ Glucose tolerance in EMPA vs. NCD	↓ Total cholesterol (^∗∗∗^) ↓ LDL‐C (^∗∗∗^) ↔ TG ↔ HDL‐C	Not reported	↓ Body weight with semaglutide vs. HFD (^∗∗∗∗^) ↔ Glucose tolerance in semaglutide vs. NCD ↔ Glucose tolerance in semaglutide vs. EMPA ↓ Total cholesterol with semaglutide vs. HFD (^∗∗∗^) ↓ TG with semaglutide vs. HFD (^∗∗^) ↓ LDL‐C with semaglutide vs. HFD (^∗^) ↔ HDL‐C with semaglutide vs. HFD ↔ Lipid profile parameters in semaglutide vs. EMPA ↔ Rate of glucose increases and decline patterns in semaglutide and EMPA groups vs. NCD (similar temporal glucose kinetics)

Miklankova et al. [43]	↓ Body weight in Wistar (^∗∗^) and HHT (^∗∗^) rats	↓ Visceral fat in Wistar (^∗∗^) and HHT (^∗∗^) rats	↓ Nonfasting blood glucose in HHTg rats (^∗∗∗^) ↔ Nonfasting blood glucose in Wistar rats ↓ Insulin levels in Wistar (^∗^) and HHT (^∗∗∗^) rats	↓ Serum triacylglycerol in HHTg rats (^∗∗∗^); ↔ in Wistar rats ↓ Palmitoleic acid levels (^∗^–^∗∗∗^) and dihomo‐γ‐linoleic acid levels (^∗^–^∗∗∗^) ↑ n‐3 PUFA levels (^∗^–^∗∗∗^) and DHA levels (^∗^–^∗∗∗^) ↓ Myocardial triacylglycerol (^∗^–^∗∗∗^), diacylglycerol (^∗^–^∗∗∗^) and lysophosphatidylcholine levels (^∗^–^∗∗∗^) ↑ Myocardial phosphatidylcholine/phosphatidylethanolamine ratio	↓ hepatic Scd‐1 mRNA expression (^∗^–^∗∗∗^) and D9D index (^∗^–^∗∗∗^) ↑ hepatic linoleic acid levels (^∗^–^∗∗∗^)	↑ glucagon levels in Wistar rats (^∗^); ↔ in HHTg rats ↑ food intake (7% increase in EMPA‐treated groups, ^∗^) ↑ IL‐10 levels in Wistar (^∗∗^) and HHTg (^∗^) rats ↓ hs‐CRP levels in Wistar rats (^∗∗^)

Zambrano‐Vásquez et al. [44]	↓ Body weight with EMPA + Met combination vs. MASLD group (^∗^); ↔ with EMPA monotherapy	Not reported	↓ Fasting blood glucose (EMPA + Met and EMPA alone vs. MASLD, ^∗^) ↑ Glucose tolerance: ↓ OGTT AUC with EMPA + Met vs. MASLD (^∗^) and vs. monotherapies (^∗^)	↓ Plasma TG (all treatments vs. MASLD, ^∗^) ↓ Total cholesterol (EMPA + Met only vs. MASLD, ^∗^) ↑ HDL‐C (EMPA + Met vs. MASLD, ^∗^) ↓ Hepatic TG (EMPA + Met vs. MASLD); ↔ hepatic cholesterol (trend only)	↓ ALT, AST, ALP, GGT, total bilirubin, direct bilirubin (all treatments vs. MASLD, ^∗^; combination most effective for ALP, GGT) ↓ Liver weight and liver weight/body weight ratio (combination most effective, ^∗^) ↓ Hepatic steatosis and % damaged hepatocytes (^∗^) ↓ NASH activity scores (apoptosis, ballooning, inflammation; combination most effective) ↓ Hepatic lipid droplets (^∗^) ↓ MDA/lipid peroxidation (all treatments, ^∗^) ↑ Mitochondrial Complex I and II activity (all treatments, ^∗^) ↑ GPx and SOD activity (combination most effective, ^∗^) ↓ Nuclear ChREBP and SREBP‐1c expression ↑ Nrf2 nuclear expression (combination vs. MASLD, ^∗^)	Modulation of Nrf2‐mediated antioxidant pathway No hypoglycemia detected; treatments well tolerated

Berger et al. [45]	↓ Body weight (^∗^)	Not reported	↔ Fasting blood glucose (trend ↓) ↓ Hyperinsulinemia and HOMA‐IR (^∗^)	Not reported	↓ Liver congestion (trend ↓ liver weight)	↓ left ventricle hypertrophy and normalized biventricular weight (^∗^) ↔ left ventricle ejection fraction and most diastolic parameters Trend ↓ left atrial mass ↑ exercise capacity (^∗^)

Hojná et al. [46]	↓ Body weight gain in both empagliflozin‐treated groups (^∗∗^); greater reduction with ACEi + EMPA group	↓ Epididymal fat mass with ACEi + EMPA group (^∗^); ↔ with Triple + EMPA group	ACEi + EMPA group: ↑ glucose tolerance and ↓ insulin resistance (↓ nonfasting glucose, ↓ plasma insulin, ↓ HOMA‐IR, improved OGTT AUC; ^∗∗^) Triple + EMPA worsened glucose/insulin parameters	↔ Circulating lipids (TAG, cholesterol, HDL‐C) ACEi + EMPA group: ↓ hepatic cholesterol; ↓ ectopic lipid accumulation (liver, kidney, heart, skeletal muscle TG) (^∗^–^∗∗^); Triple + EMPA group: ↑ ectopic lipids	ACEi + EMPA: ↓ hepatic steatotic gene expression (SREBP, FASN, ACACA) and lipogenic markers (AKT, SREBP, PGC‐1α mRNA); Triple + EMPA group: disrupted lipogenic gene regulation	—

Alharbi et al. [47]	Not reported	Not reported	↓ Fasting serum glucose (^∗^), insulin (^∗^), and HOMA‐IR (^∗^)	↓ Serum TG, TC, LDL‐C, VLDL‐C (^∗^), and FFA (^∗^)↑ serum HDL‐C (^∗^)	↓ Serum ALT, AST (^∗^), hepatic steatosis/necrosis/inflammation scores (^∗^), ↓ hepatic MDA, 4‐HNE, and ↑ hepatic GSH, GPX4, Nrf2 (^∗^)↓ serum iron; ↑ hepatic transferrin, hepcidin (^∗^)↓ hepatic NCOA4; ↑ FTH1 expression (^∗^)	↓ Hepatic calcineurin A, calcium (^∗^)↓ hepatic Beclin‐1; ↑ LC3‐II, ↓ p62/SQSTM1 (autophagy modulation, ^∗^)↓ hepatic NF‐κB, IL‐6, TGF‐β1 (^∗^)↓ hepatic FABP1, PPAR‐γ, CD36 expression (^∗^)

*Note:* Symbols: ↓ indicates a significant decrease, ↑ indicates a significant increase, ↔ indicates no significant change, Δ denotes change, *p* (*p*‐value), and > indicates a greater effect. *p* values are presented as: ^∗^ *p* < 0.05; ^∗∗^ *p* < 0.01; ^∗∗∗^ *p* < 0.001; ^∗∗∗∗^ *p* < 0.0001. EMPA = empagliflozin, Met = Metformin, Dio2 = Type II iodothyronine deiodinase, HOMA‐IR = homeostatic model assessment for insulin resistance, IL‐1β = interleukin‐1 beta, i.c.v. = intracerebroventricular, ALT = alanine aminotransferase, AST = aspartate aminotransferase, ALP = alkaline phosphatase, SOD = superoxide dismutase, AUC = area under the curve, IL‐10 = interleukin‐10, Scd‐1 = stearoyl‐CoA desaturase‐1, DHA = docosahexaenoic acid, PUFA = polyunsaturated fatty acids, TAGs = triacylglycerols, LPC = lysophosphatidylcholine, NRF1 = nuclear respiratory factor 1, SIRT7 = sirtuin 7, CCL2 = chemokine (C–C motif) ligand 2, NOS2 = nitric oxide synthase 2, CAT = catalase, Col1a1 = collagen Type I alpha 1 chain, TIMP1 = tissue inhibitor of metalloproteinase 1, PC = phosphatidylcholine, PI = phosphatidylinositol, LPE = lysophosphatidylethanolamine, NASH = nonalcoholic steatohepatitis, MDA = malondialdehyde, GPx = glutathione peroxidase, ChREBP = carbohydrate‐responsive element–binding protein, SREBP‐1c = sterol regulatory element–binding protein‐1c, FASN = fatty acid synthase, AKT = protein kinase B, PGC‐1α = peroxisome proliferator–activated receptor gamma coactivator 1‐alpha, GSH = reduced glutathione, 4‐HNE = 4‐hydroxynonenal, Nrf2 = nuclear factor erythroid 2–related factor 2, NCOA4 = nuclear receptor coactivator‐4, FTH1 = ferritin heavy chain 1, LC3‐II = light chain 3‐II, p62/SQSTM1 = sequestosome 1, NF‐κB = nuclear factor kappa‐light‐chain enhancer of activated B cells, FABP1 = fatty acid–binding protein‐1, PPAR‐γ = peroxisome proliferator–activated receptor gamma, CD = cluster of differentiation, FGF21 = fibroblast growth factor 21, hs‐CRP = high‐sensitivity C‐reactive protein, TG = triglycerides.

Abbreviations: α‐SMA = alpha‐smooth muscle actin, BAT = brown adipose tissue, FFA = free fatty acids, FFD = fast food diet, GGT = gamma‐glutamyl transferase, HDL = high‐density lipoprotein, HFD = high‐fat diet, LDL‐C = low‐density lipoprotein cholesterol, NAFLD = nonalcoholic fatty liver disease, NAS = NAFLD activity score, NEFA = nonesterified fatty acids, NFD = normal‐fat diet, OGTT = oral glucose tolerance test, TNF‐α = tumor necrosis factor alpha, VAT = visceral adipose tissue, WAT = white adipose tissue.

**FIGURE 3:**
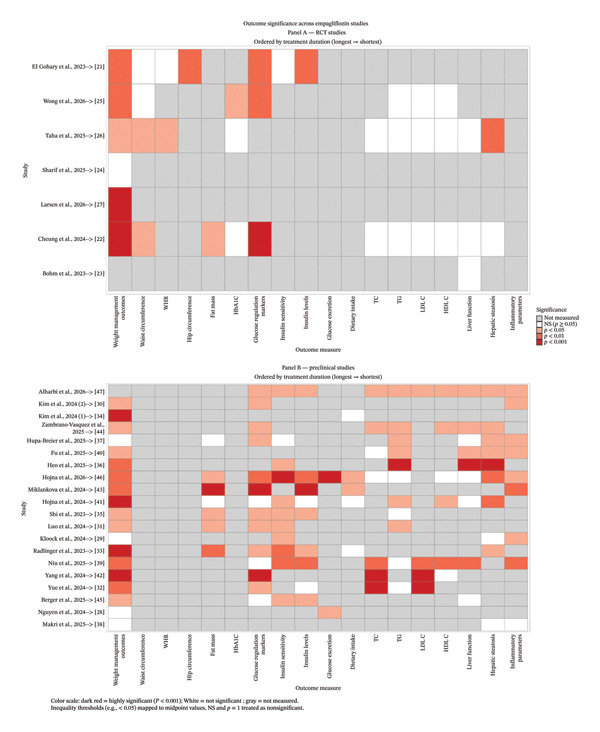
Heatmap illustrating the outcomes assessed and their statistical significance across included studies in this systematic review. Each row represents an individual study, and columns denote specific outcome domains, including anthropometric, glycemic, lipid, dietary, inflammatory, and hepatic parameters. Color intensity corresponds to statistical significance, with deeper red indicating significant findings, white representing nonsignificant results, and gray denoting outcomes not assessed. Studies outlined with a blue border represent randomized controlled trials, whereas those without a border are preclinical studies. Abbreviations: HDL_C, high‐density lipoprotein cholesterol; LDL_C, low‐density lipoprotein cholesterol; TC, total cholesterol; WHR, waist‐to‐hip ratio.

Figure 3 compares the outcomes assessed across the included studies, highlighting the frequency and statistical significance of findings.

Among the seven human RCTs, six assessed weight management outcomes, of which five reported statistically significant improvements. Waist circumference was evaluated in four RCTs, with two demonstrating significant reductions. Most RCTs demonstrated improved glucose‐regulation markers. However, insulin‐related outcomes were evaluated infrequently: One study reported a significant reduction in insulin levels, whereas insulin sensitivity (HOMA‐IR) was not improved. Two RCTs evaluated hepatic steatosis and one study reported significant improvements, whereas three RCTs assessed liver function parameters and none of them reported statistically significant reductions in liver enzymes. Lipid parameters were evaluated in four RCTs, and no study showed significant changes.

Across the 20 animal studies, EMPA consistently reduced body weight and fat mass. Markers of glycemic control, insulin sensitivity, and insulin levels were also frequently improved, although the significance of changes varied across experimental models. Several experiments also demonstrated reductions in total cholesterol and LDL‐C. Improvements in hepatic steatosis were reported in eight of 10 studies evaluating this outcome. Furthermore, inflammatory markers improved significantly in eight of nine studies.

### 3.4. RoB in Included Studies

**FIGURE 4:**
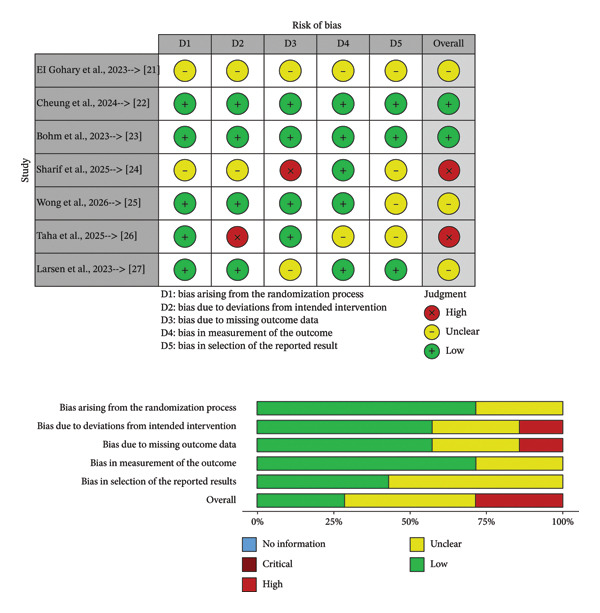
Cochrane risk‐of‐bias assessment in selected studies: distribution of methodological quality across different evaluated domains. Visualization generated using the robvis tool.

Figure 4 presents the distribution of methodological quality using Cochrane RoB assessment for the reviewed human studies: two out of seven studies [22, 23] were assessed with a low risk across all domains. Three studies [21, 25, 27] were judged to have some concerns. In contrast, two studies [24, 26] were assessed as having a high overall RoB. Across the included RCTs, the domains related to the deviations from intended interventions (D2) and missing outcome data (D3) showed the greatest variability, whereas bias in randomization process (D1) and outcome measurement (D4) was generally judged to be low risk.

**FIGURE 5:**
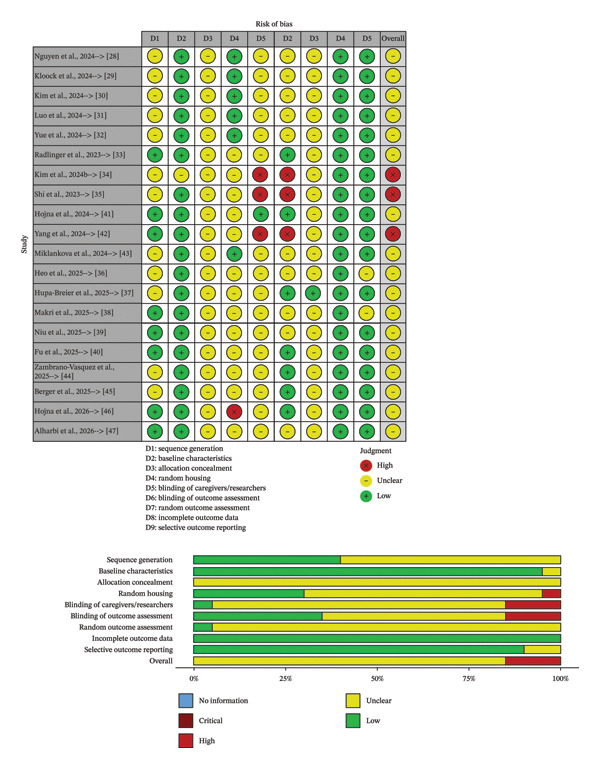
SYRCLE’s risk‐of‐bias assessment in selected studies: distribution of methodological quality across different evaluated domains. Visualization generated using the robvis tool.

As shown in Figure 5, the SYRCLE RoB assessment of the 20 included animal studies revealed that key procedural domains were predominantly rated as unclear: Allocation concealment was unclear in all 20 studies, and random outcome assessment was unclear in all 19 studies; blinding of caregivers/researchers was unclear in 16 studies (three of which were rated as high risk); and blinding of outcome assessment was unclear in 10 studies (three high risk). In contrast, baseline characteristics, incomplete outcome data, and selective outcome reporting were largely rated as low risk. Overall, 17 studies were assessed as having an unclear RoB and three as having a high RoB.

## 4. Discussion

Based on the reviewed studies, the clinical evidence regarding EMPA use in nondiabetic individuals with obesity remained limited but suggested modest beneficial effects. Across the available RCTs, EMPA showed generally positive effects, including decreases in body weight and fat mass, as well as enhancements in glucose levels. However, the evidence for better control of liver enzymes, hepatic steatosis, and lipid parameters remained limited and inconsistent.

In contrast, a larger body of evidence from experimental animal models has reported broader metabolic effects of EMPA, including reductions in body weight and adiposity, as well as enhancement of glycemic control, insulin sensitivity, hepatic steatosis, and selected lipid parameters. Improvements in inflammatory markers were also observed in several preclinical studies. However, EMPA did not consistently affect liver enzyme levels; additionally, findings regarding lipid parameters remained inconsistent across studies. These differences highlight the early and heterogeneous nature of the current evidence base and underscore the need to interpret preclinical findings cautiously when translating them to clinical settings.

When interpreting these findings, several translational and methodological factors may explain the differences between animal models and human clinical trials. Animal studies typically administered much higher weight‐adjusted doses (10–30 mg/kg/day) compared to the fixed doses used in human trials (10–12.5 mg/day). These higher exposures may have contributed to the more pronounced metabolic effects observed in preclinical studies. Moreover, in contrast to the more heterogeneous patient populations observed in human trials, animal models were generated under tightly controlled dietary and environmental conditions, hence enhancing therapeutic efficacy. The treatment efficacy is also influenced by species‐specific variations in glucose metabolism, energy use, and lipid metabolism [43]. These disparities highlight the necessity for better‐designed clinical trials to improve the applicability of animal study findings to human conditions.

### 4.1. EMPA’s Role in Weight Reduction

Weight loss by behavioral modifications is difficult for those with recurrent overeating, poor motivation, and inadequate self‐discipline [48]. Moreover, maintaining weight loss is particularly challenging, with only approximately 15% of individuals able to sustain at least a 10% reduction without surgical intervention or medication [49, 50]. Weight reduction aids in diabetes management due to the association between obesity and the condition. Research demonstrates that individuals with Type 2 diabetes may have a weight loss of 1.86 kg at a dosage of 10 mg and 1.95 kg at a dosage of 25 mg, respectively [51]. A potential reason for the observed weight loss is elevation in core body temperature and basal metabolic rate [52, 53]. In 2019, a randomized trial by Javed et al. evaluated the impacts of EMPA and metformin on the body measurements and metabolic parameters of women with PCOS. Similar to EI Gohary et al., in the EMPA cohort, both hip and waist circumferences exhibited significant reductions after 12 weeks, while BMI remained unchanged [54]. More recently, Sharif et al. also evaluated EMPA in women with PCOS and reported significant reductions in body weight and BMI after 24 weeks of treatment, with greater improvements than those observed with metformin. Differences in comparator groups may elucidate the discrepancy. After 12 weeks of therapy, BMI, hip circumference, and fat mass significantly increased in the metformin group, in contrast to the findings of El Gohary et al. regarding the combined effects.

In animal models, EMPA significantly mitigated diet‐induced weight gain, resulting in comparable body weights for mice in the WDE group and those on a control diet. This is probably due to the mechanisms of SGLT2 inhibitors that enhance urinary energy loss and boost energy expenditure [53]. However, as the SYRCLE assessment revealed, blinding procedures were frequently unclear in the majority of animal studies, raising potential concerns regarding detection bias in these weight outcomes. People frequently lose less weight than expected despite using SGLT2 inhibitors, as glycosuria has been shown to cause caloric loss [55]. A higher calorie intake may make up for this disparity, according to mathematical models, but human investigations have failed to find any indication of an increase in appetite or caloric consumption [56–58]. Consistent with these observations, Wong et al. reported significant weight loss without changes in caloric intake, whereas preclinical studies have shown mixed effects on food consumption despite generally favorable effects on body weight. Resting energy expenditure drops as people lose weight on a diet, perhaps because their energy balance is negative. Glycosuria following SGLT2 inhibitor use may cause similar metabolic changes, while the precise molecular pathways are still unknown [59]. Certain individuals using SGLT2 inhibitors may encounter heightened appetite, resulting in increased food intake and thereby hindering weight loss [60]. Kim et al. likewise discovered that EMPA initially stimulated appetite‐regulating genes; however, this effect diminished after 3 weeks. Accordingly, the impact of EMPA on gene expressions was dose‐dependent; a larger increase in appetite‐suppressing gene expression was observed at higher dosages. However, Radlinger et al., who utilized three times the dose of EMPA as Kim et al. (30 vs. 10 mg/kg/day), discovered no significant change in daily food intake across research groups, while water intake was higher in EMPA‐treated mice. Hojná et al. also observed no disparity in food intake between the groups administered a high‐fat diet and those receiving EMPA. The EMPA‐treated group exhibited a drop in body weight relative to the untreated SHR + HF group beginning at Week 5 of treatment, with a final weight gain difference of 17 ± 3 g (*p* < 0.01), similar to the delayed weight loss reported by Kim et al. However, the delayed effect of EMPA on body weight may be due to the animals’ age at the end of the experiment, which typically decelerates weight gain compared to younger rats. Notably, even after 6 months of treatment, Makri et al. observed no statistically significant difference in weight gain between the groups of mice subjected to a fast‐food diet, whether untreated or treated with EMPA.

### 4.2. EMPA’s Effects on Insulin and Glucose Regulation

The secretion of nonesterified fatty acids, proinflammatory cytokines, and adipocytokines from adipose tissue leads to insulin resistance, Type 2 diabetes, and hepatic steatosis [61]. EMPA may produce effective glycemic control, either as monotherapy or in combination with other medications. Shi et al.’s study found that EMPA treatment led to significant weight reduction, reducing free fatty acids, HOMA index, fasting insulin levels, and fat mass. Consistently, Radlinger et al. reported EMPA prevented excessive weight gain in Western diet mice, although it did not impact adipose tissue depots in control diets. MRI analyses further showed EMPA reduced diet‐induced adipose tissue expansion. Similarly, EMPA therapy reduced the amount of epididymal fat adipocytes in C57BL/6 mice, according to research by Vallon et al. Conversely, in insulin‐deficient Akita mice, an increase in adipocyte size was noted [62]. A 24‐week trial that included a placebo control demonstrated that doses of 10 mg and 25 mg led to significant reductions in HbA1c levels of −1.43% and −1.44%, respectively, compared to the placebo [63]. In contrast to El Gohary et al., Javed et al. found no notable alterations in insulin sensitivity in the EMPA or metformin groups following 12 weeks of treatment [54]. A 2014 meta‐analysis conducted by Liakos et al. demonstrated that EMPA was more effective than metformin at lowering blood glucose levels and enhancing fasting insulin in diabetic patients, encompassing 10 studies with 6203 participants [64]. Radlinger et al. and Miklankova et al. found a strong link between body weight and whole‐body insulin sensitivity. They correlated body weight with glucose infusion rate (GIR) and found that WDE‐fed mice required significantly higher GIR to maintain sustained euglycemia under hyperinsulinemic conditions compared to WD‐fed mice. Mice fed either WDE or CD had comparable GIR and insulin sensitivity. After controlling for changes in lean body mass and plasma insulin levels under clamp conditions, the difference in insulin sensitivity became even more apparent. Despite the lack of control for lean body mass, insulin sensitivity in WDE‐fed mice increased by mitigating excessive weight gain. Moreover, Radlinger et al. observed that insulin receptor expression levels remained consistent across all groups. Evidence suggests that while EMPA is effective in lowering overall blood glucose levels, its impact varies depending on the specific format of glucose assessment. Yue et al. and Kim et al. found that EMPA has a significant effect on blood glucose levels, but no significant change in fasting glucose levels. Shi et al. and Hojná et al. observed that EMPA reduced blood glucose levels after insulin injection and glucose administration but did not likewise affect fasting glucose levels. Similar findings were reported by Niu et al. and Berger et al., who observed improvements in glucose tolerance, insulin resistance, and hyperinsulinemia despite nonsignificant changes in fasting glucose levels. Conversely, Hupa‐Breier et al. reported significant reductions in fasting glucose without corresponding improvements in HOMA‐IR. Despite these improvements, according to the study by Hojná et al., EMPA did not alleviate insulin resistance that developed in skeletal muscle and visceral adipose tissue as a result of HFD, nor did it reverse the increase in serum insulin levels. In animal studies, while many reported improvements in glycemic parameters, the frequent lack of blinding, allocation concealment, and random outcome assessment necessitates a cautious interpretation of these findings. The observed improvements may partly reflect indirect effects via weight reduction, which was strongly correlated with insulin sensitivity across several models.

### 4.3. EMPA as a Potential Treatment for MASLD

Reducing hepatic steatosis could provide health advantages even in the absence of MASH (metabolic dysfunction–associated steatohepatitis), as it elevates the likelihood of cardiovascular disease, cancer, and serious infections such as COVID‐19 [65–68]. Böhm et al. also found a correlation between worse outcomes, increased risk of heart failure hospitalization, and cardiovascular‐related death, but no association was found between ALT/AST levels and adverse cardiac outcomes. MASLD is currently treated with vitamin E for people without diabetes and pioglitazone for patients with diabetes, but both have significant risks, including increased prostate cancer risk and all‐cause mortality [69, 70]. Pioglitazone, on the other hand, is associated with increased body weight, edema, congestive heart failure, reduced bone mineral density, and bladder neoplasia, making it a second‐line treatment option [71]. Weight loss of 3.0% to 7.0% can reduce liver inflammation and steatosis, potentially leading to hepatic steatosis resolution [72]. Research indicates that a weight decrease above 10% of one’s initial weight considerably facilitates the resolution of steatosis in individuals affected by obesity [73, 74]. The study identified SGLT2 inhibitors as a possible therapy for MASLD in individuals with Type 2 diabetes. The E‐LIFT trial observed a 4.9% decrease in magnetic resonance imaging proton density fat fraction (MRI‐PDFF) following 20 weeks of treatment with EMPA [75], while another study showed a 2.3‐fold reduction in liver fat content after 24 weeks [76]. This could be due to EMPA’s ability to reduce oxidative stress and alleviate inflammation, making it a promising treatment option [77]. The −1.02% reduction seen in the study by Cheung et al. is less than the −1.8% to −3.74% reductions reported in other RCTs examining the effects of SGLT‐2 inhibitors on hepatic fat levels in individuals with diabetes [75, 76, 78, 79]. Factors such as a population without diabetes, with lower initial liver fat content, and a lower dose of EMPA in the study by Cheung et al. may explain these variations. To determine whether EMPA is effective in treating MASH in nondiabetic individuals, further studies are required, especially at the higher dosage of 25 mg daily. Cheung et al.’s study found that EMPA did not significantly impact MASH resolution, unlike previous studies. Cheung et al.’s study primarily consisted of individuals without MASH (baseline ALT levels 31 U/L vs. > 47–80 U/L), suggesting that this variance may be attributable to differences in patient populations. Böhm et al. also observed no notable differences in ALT and AST levels between the groups receiving the placebo and those treated with EMPA after nearly 2 years of EMPA treatment, likely due to the exclusion of patients with elevated liver values.

Among the animal studies, Kloock et al. did not observe a significant impact of EMPA on liver weight or MASLD scores. One possible explanation for this discrepancy is that the study population exhibited only mild signs of insulin resistance, which may not be comparable to full‐blown T2DM. Additionally, any potential benefits on liver steatosis might have been offset by the elevated NEFA levels observed in their study. Although EMPA did not affect circulating NEFAs, it altered the fatty acid composition of the NEFA lipid class, which is likely crucial for fatty acid transport and uptake in the myocardium [43]. Radlinger et al. induced overt NAFLD in WD‐fed mice by administering EMPA at a higher dose than previously studied. They found that WDE‐fed mice had lower degrees of hepatic steatosis and higher hepatic insulin signaling compared to WD‐fed mice. More recent animal studies have largely corroborated these findings, demonstrating improvements in hepatic steatosis, fibrosis, oxidative stress, inflammation, and, in several cases, liver enzyme levels following EMPA treatment [36, 37, 39, 40], although Makri et al. reported no significant histological improvement after 6 months of treatment in a fast‐food diet mouse model. Blood transaminase remained unchanged in the reviewed animal studies, but these markers are not reliable screening indicators for MASLD since 80% of patients with MASLD exhibit normal ALT levels [80].

### 4.4. Effects of EMPA on Lipid Metabolism

A meta‐analysis involving patients with Type 2 diabetes indicated that SGLT2 inhibitors, even when administered alone, might have a slight effect on the serum lipid profile [81]. According to Yue et al., EMPA was effective in improving serum cholesterol. Like Yang et al., their findings indicated that EMPA lowered serum cholesterol and LDL‐C levels. This increase has been linked to a decrease in the clearance of LDL‐C from the bloodstream [82]. Both Yue et al. and Yang et al. applied the same fat composition in their dietary regimen and maintained a consistent HFD duration in their studies. However, Miklankova et al. found no significant effect of EMPA on serum cholesterol in a hereditary hypertriglyceridemic model. Like previously [83], Yang et al.’s findings indicated no significant changes in HDL‐C levels and function influenced by EMPA. Like Shi et al., Yang et al., and Rau et al. [84], their study found that EMPA treatment did not significantly affect TG levels, unlike the study by Miklankova et al. that found improvements in serum dyslipidemia by reducing triglyceride levels. Consistent with other studies, Miklankova et al. observed no significant change in HDL‐C levels, as per previous research. More recent studies have provided additional evidence supporting favorable lipid effects of EMPA. Niu et al. observed reductions in total cholesterol and LDL‐C together with increased HDL‐C levels, while Alharbi et al. reported improvements in all lipid parameters. Similarly, Zambrano‐Vásquez et al. demonstrated reductions in plasma triglycerides and improvements in HDL‐C, particularly when EMPA was combined with metformin. Yue et al.’s and Miklankova et al.’s studies on TG results differed due to differences in study populations. Miklankova et al. examined hereditary hypertriglyceridemic subjects, while Yue et al. focused on younger mice with obesity, induced by a 12‐week high‐fat diet. Miklankova et al. found that EMPA’s influence on lipid metabolism, particularly in lipid metabolite formation, was more substantial in the heart than in the liver. More recent studies have further suggested that EMPA may exert important effects on hepatic lipid metabolism, including reductions in hepatic triglyceride accumulation and lipogenic gene expression, although some findings remain model‐dependent. The animal studies generally reported favorable changes in lipid profiles, though the prevalence of unclear risk in most domains casts uncertainty on the objectivity of these measurements.

Among studied RCTs, serum lipid parameters were evaluated by four studies. None of them have reported significant improvements in lipid parameters. The absence of significant lipid changes may partly reflect the metabolic characteristics of the included populations. For example, although participants in the study by Cheung et al. had MASLD, their baseline lipid profiles were largely within normal ranges, despite confirmed hepatic steatosis on MRI‐PDFF, indicating relatively mild systemic dyslipidemia in this nondiabetic population. This finding highlights the varied metabolic characteristics of MASLD, where the buildup of fat in the liver can happen without major changes in serum lipid levels, especially in people who do not have diabetes or severe metabolic disorders [65–68]. Additionally, the study population consisted of nondiabetic individuals with moderate overweight (mean BMI approximately 27 kg/m^2^), suggesting less severe insulin resistance and lipid dysregulation compared with populations with obesity or Type 2 diabetes. EMPA might mainly enhance the management of intrahepatic lipids and decrease the buildup of hepatic fat instead of directly affecting the levels of lipids in circulation [22, 75]. Similar findings were reported by Wong et al. and Taha et al., who observed no significant changes in circulating lipid parameters despite beneficial effects on weight‐related and hepatic outcomes. Notably, Wong et al. studied overweight individuals with schizophrenia spectrum disorders, while Taha et al. enrolled patients with MAFLD and established hepatic fibrosis. In contrast, Sharif et al. reported significant improvements in all measured lipid parameters in women with PCOS, suggesting that the lipid effects of EMPA may vary according to the underlying metabolic profile and severity of dyslipidemia in the study population.

### 4.5. Underlying Mechanisms of EMPA

**FIGURE 6:**
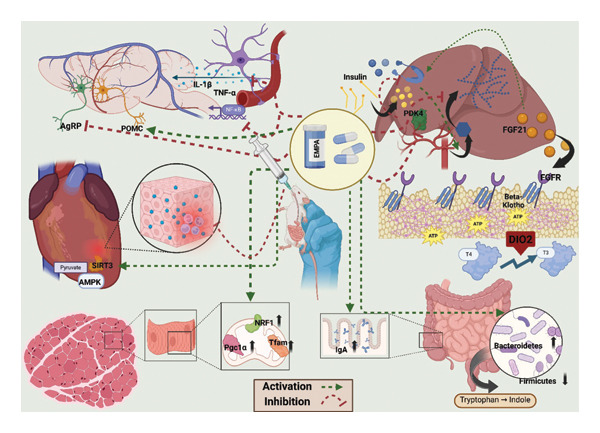
How EMPA works: key mechanisms from the reviewed studies. Abbreviations: TNF‐α, tumor necrosis factor alpha; IL‐1β, interleukin‐1 beta; AgRP, agouti‐related peptide; POMC, proopiomelanocortin; NF‐κB, nuclear factor kappa B; Tfam, mitochondrial transcription factor A; Pgc‐1α, peroxisome proliferator–activated receptor gamma coactivator 1‐alpha; EMPA, empagliflozin; FGF21, fibroblast growth factor 21; FGFR, FGF receptor; AMP, adenosine monophosphate; SIRT3, sirtuin 3; PDK4, pyruvate dehydrogenase kinase 4; ATP, adenosine triphosphate; AMPK, AMP‐activated protein kinase; DIO2, iodothyronine deiodinase Type II; T3, triiodothyronine; T4, thyroxine; NRF1, nuclear respiratory factor 1. Created with BioRender.com.

The rise in obesity and T2DM requires better knowledge of metabolic alterations. This will help create SGLT2 inhibitor‐based weight loss regimens for T2DM patients, improve glycemic control, and reduce related comorbidities. Figure 6 illustrates the underlying mechanisms of EMPA as identified in the reviewed studies. Most of the mechanistic insights regarding the metabolic effects of EMPA in nondiabetic obesity are derived primarily from preclinical animal studies. Therefore, the mechanisms discussed below should be interpreted as potential biological pathways suggested by experimental models rather than mechanisms that have been conclusively demonstrated in humans.

#### 4.5.1. FGF21 and Its Role in Metabolism

Prolonged exposure to the metabolic hormone FGF21 may improve central nervous system performance due to its intricate mechanisms associated with heightened energy expenditure and weight management [85]. Consistent with previous studies in both animals and humans [52, 86, 87], Nguyen et al. showed that mice fed a 60% high‐fat diet had higher FGF21 hormone levels which EMPA partially protected, indicating that the EMPA treatment group did not experience significant weight change. Reduced FGF21 receptors, or “FGF21 resistance,” may occur in the context of a high‐fat diet, which in turn can cause plasma FGF21 levels to rise to compensate [86]. Although there was no clear alteration in mRNA levels, Nguyen et al. noted that adipose tissue FGF receptor levels were lower in mice that were administered HFD. Additionally, the study found that FGF receptor expression was lower in white adipose tissue of HFD‐fed mice, and its mRNA levels slightly increased in mice receiving EMPA. Consequently, these changes did not significantly impact the hypothesis that EMPA counteracts HFD‐induced FGF21 resistance or restores normal peptide levels. Despite reduced levels of FGF21 protein in the hepatic tissue of mice fed a diet rich in fat, liver mRNA levels of FGF21 remained constant across all animal groups. This also suggests that FGF21 secretion from the liver may be elevated in the HFD group. HFD‐fed mice showed reduced mRNA levels of YipF6, a protein responsible for sequestering FGF21 in hepatocytes. However, western blot analysis and YipF6 findings did not clarify the difference in serum FGF21 levels between HFD and HFD + EMPA groups or indicate that EMPA influenced FGF21 liver secretion. Reduced FGF21 levels in the EMPA group could be attributable to accelerated FGF21 clearance or degradation. Interestingly, Hupa‐Breier et al. likewise reported reduced hepatic FGF21 expression following EMPA treatment, accompanied by improvements in hepatic steatosis, fibrosis, inflammation, and oxidative stress despite no significant changes in body weight. Hence, further research is required [88]. Thyroid hormone conversion from T4 to T3 is facilitated by the deiodinase enzyme Dio2, which is increased by FGF21 [86]. Nguyen et al. found that Dio2 levels were elevated in white adipose tissue of the HFD group but returned to baseline in EMPA ‐treated mice. Considering that decreased Dio2 expression results in decreased T4 to T3 conversion, this provides evidence of a connection between EMPA, FGF21, and adipocyte metabolism [89]. Preclinical studies suggest that modulation of FGF21 signaling may contribute to the cardiometabolic effects observed with SGLT2 inhibitors [90]. In contrast, Nguyen et al. found no effect of EMPA on the cardiac FGF21 expression in HFD‐fed mice, although the reduction in systemic FGF21 levels may still support heart health.

#### 4.5.2. Weight Loss and PDK4 Methylation Reversal

Experimental research has linked hepatic PDK4 expression to diminished insulin sensitivity and the onset of fatty liver development via changes in fatty acid metabolism [91]. EMPA may mitigate the impact of weight reduction on PDK4 expression, as prior studies have shown that alterations in PDK4 promoter methylation caused by obesity had been reversed after substantial weight loss [92]. Additionally, Radlinger et al. also discovered that WDE‐fed mice had reduced fatty acid uptake due to a reduction in Pdk4 mRNA expression. Their findings of improved insulin sensitivity and increased gluconeogenesis raise the possibility that the increased hepatic gluconeogenesis in WDE‐fed mice is due to this decrease. Decreased carbohydrate oxidation, increased fatty acid oxidation, and gluconeogenesis may occur when Pdk4 mRNA levels are low [93]. Conversely, a downregulation of glucokinase mRNA in WDE‐fed mice suggests reduced glycolysis, potentially enhancing hepatic insulin sensitivity. Skeletal muscle glucose disposal is crucial for systemic insulin sensitivity, with impaired mitochondrial function contributing to cellular insulin resistance. It has been hypothesized that EMPA may influence the morphology and dimensions of mitochondria, therefore enhancing citrate synthase activity and the mRNA expression of Pgc1α, Nrf1, and Tfam. This subsequently leads to an increase in the size and roundness of mitochondria in skeletal muscle [94].

#### 4.5.3. Hypothalamic Inflammation and Metabolic Dysfunction

The hypothalamic neurocircuitry, a sophisticated network of neuropeptides, regulates energy balance and may have a role in obesity [95], but the precise processes influencing this regulation remained ambiguous. The hypothalamic melanocortin system governs energy balance by stimulating hunger via AgRP neurons in the arcuate nucleus and suppressing it through POMC neurons [96, 97]. Kim et al. (1) found that EMPA, when administered directly into the mouse brain, modulated appetite‐related and energy metabolism genes, potentially influencing body weight regulation. Their findings highlight its physiological effects in obesity‐induced metabolic syndrome, as they observed an increase in AgRP mRNA expression at 3 and 6 h postadministration and a substantial rise in POMC mRNA expression after 3 weeks. The development of metabolic diseases may be accelerated when there is an excess of energy, which causes inflammation in the hypothalamus, disruptions in insulin and leptin communication, and dysfunction in neurons. This, in turn, causes astrocyte accumulation and activation [98, 99]. Kim et al. (2) conducted an experiment on the impact of EMPA on hypothalamic inflammation. Initially, EMPA had no effect on reactive astrocytes or GFAP expression. However, after 3 weeks of HFD, both increased significantly. By the 16th week, EMPA effectively reduced the rise in reactive astrocytes. Despite no significant changes in cytokine expression after 3 days, EMPA significantly decreased high concentrations of IL‐1β and TNF‐α after 3 weeks, suggesting EMPA’s role in modulating astrocyte activation and cytokine expression. Experimental studies indicate that chronic HFD exposure has the potential to activate the inflammatory NF‐κB signaling pathway. Additionally, targeting NF‐κB inhibition specifically in neurons or glial cells might diminish astrocyte activation and facilitate weight loss [100]. Similarly, in the study by Kim et al. (2), elevated glucose levels triggered the nuclear translocation of cytosolic NF‐κB P65, a process that EMPA partially inhibited. Following high‐glucose treatment, the number of p‐P65‐positive cells in astrocyte nuclei increased significantly. However, EMPA regulated the NF‐κB signaling pathway by diminishing glucose‐induced P65 nuclear translocation. While evidence indicates the reduction of NF‐κB signaling in astrocyte cultures, further research is required to investigate the roles of glial cells in animal models.

#### 4.5.4. Role of SIRT3 in Cardiac Function and Obesity

EMPA has shown efficacy in improving cardiac dysfunction linked to obesity [101–103]. SIRT3 is a pivotal metabolic regulator that significantly influences cellular metabolism and obesity treatment. It regulates energy metabolism, oxidative stress management, and cellular autophagy. However, in obesity, SIRT3 activity declines in cardiac tissue, increasing cardiomyocyte vulnerability. Some experimental studies suggest a potential interaction between EMPA treatment and SIRT3‐mediated metabolic pathways [104]. Luo et al. found that EMPA reduces myocardial hypertrophy and fibrosis while increasing SIRT3 expression. This treatment also protects the heart from lipid metabolism disorders by modulating mitochondrial fatty acid oxidation in cardiac tissue [105]. Aortic vascular smooth muscle cells are essential for fatty acid β‐oxidation, providing energy for their proliferation and migration. The multistep process of adductor fat production involves many enzymes, including carboxylation of CoA (CoA), stearoyl‐CoA desaturase 3 (SCD3), fatty acid synthase (FASN), and long‐chain acyl‐coenzyme A synthase (ACSL), which esterifies fatty acids. This procedure produces an active acyl‐coenzyme A that is involved in the β‐oxidation of fatty acids [106, 107]. The study by Yue et al. verified that obesity induces upregulation of ACSL1 and ACSL5. This causes the aorta to produce more TG, which in turn increases the activation and β‐oxidation of free fatty acids. Additionally, endothelial and smooth muscle cells proliferate faster. However, the availability of long‐chain fatty acids as substrates for fatty acid β‐oxidation diminished owing to the reduced expression of FASN and SCD3 during the first stages of lipid synthesis induced by EMPA treatment. Furthermore, the progression of arterial lesions was delayed due to EMPA’s downregulation of ACSL1 and ACSL5, which reduced fatty acid β‐oxidation and elevated fatty acid concentrations. These findings are supported by more recent studies, showing that EMPA can suppress lipogenic pathways. Fu et al. reported reduced FASN expression following EMPA treatment, while Hojná et al. observed downregulation of several lipogenic markers, including SREBP and FASN, accompanied by reduced ectopic lipid accumulation in the liver, heart, and skeletal muscle. Miklankova et al. found that EMPA treatment positively impacted myocardial TNF‐α and IL‐10 levels, without affecting TNF‐α gene expression or circulating levels. However, the increase in the anti‐inflammatory cytokine IL‐10 levels in the myocardium was also reflected in serum levels. According to Kaur et al., TNF‐α can be produced by heart cells in both rats and mice. This indicates that the effects of EMPA might be related to the production of TNF‐α in the heart or its release from adipose tissue [108]. The amount of proinflammatory cytokines in the hearts of rats that were both overweight and diabetic was decreased when they were treated with EMPA [109]. In Type 2 diabetes patients, canagliflozin also lowered leptin and IL‐6 levels [110], while increasing anti‐inflammatory IL‐10 levels [111]. The suppression of NF‐κB and activation of AMPK, which impact inflammatory signaling pathways, may have caused the anti‐inflammatory effects. This supports Miklankova et al.’s findings [112]. Consistent with this hypothesis, Fu et al. demonstrated reductions in TNF‐α, IL‐6, and IL‐1β expression following EMPA treatment, while Alharbi et al. reported reduced NF‐κB and IL‐6 signaling together with improvements in oxidative stress markers. Although SGLT2 inhibitors can promote an anti‐inflammatory M2 subtype of macrophages, there has been little research on their effects on myocardial and cardiovascular damage [113]. EMPA may have anti‐inflammatory effects within the cardiac tissue of subjects with heart failure [114]. Miklankova et al. observed a reduction in myocardial β‐hydroxybutyrate (BHB) levels following EMPA treatment. However, it did not significantly affect circulating BHB concentrations, myocardial BHB levels, or its utilization in the heart. These findings are broadly consistent with clinical observations, showing that SGLT2 inhibitor treatment does not markedly increase circulating ketone bodies [115].

#### 4.5.5. Gut Microbiota and EMPA’s Antiobesity Effects

Obesity is linked to changes in gut microbiota, characterized by a reduction in microbial diversity and functionality [116]. Firmicutes and Bacteroidetes are commonly known as “obesity‐associated bacteria” and “lean‐associated bacteria,” respectively. An elevated ratio of Firmicutes to Bacteroidetes has been related to heightened inflammation and weight gain [117]. Individuals with obesity exhibit a diminished proportion of Bacteroidetes, although this proportion increases after weight reduction achieved through a calorie‐restricted diet [118]. Animal studies suggest that EMPA may partially restore the Firmicutes‐to‐Bacteroidetes ratio, potentially impacting gut microbiota composition [119]. Similarly, Shi et al. found that high‐fat diet groups had lower Bacteroidetes and Firmicutes abundances, but EMPA treatment counteracted these alterations, rebalancing the ratio. Tryptophan, an essential amino acid, can compromise intestinal immune function, and its deficiency makes the gut more susceptible to inflammatory diseases [120]. In order to convert tryptophan into bioactive chemicals, the gut microbiota is essential [121]. According to Shi et al., tryptophan metabolism and indole products, particularly indole‐3‐lactic acid, were found to be increased in the EMPA‐treated group, which they attributed to EMPA‐induced alterations in gut microbiota. Protecting the mucosal surface from harmful microorganisms is a crucial function of IgA. Many studies have shown how important it is for keeping the microbiota stable and gut homeostasis intact [122]. According to the findings of Shi et al., EMPA treatment stimulated IgA production, reinforcing mucosal immunity. It also affected the PPAR signaling pathway, an important regulator of energy balance, glucolipid metabolism, and inflammatory responses—all of which are disrupted in obesity [123]. However, the beneficial effects of EMPA in HFD‐fed mice were not directly dependent on gut microbiota alterations. A further analysis is required to examine the causal linkage and processes associated with EMPA’s antiobesity effects.

Despite these mechanistic insights observed in animal models, their direct translation to humans remains uncertain. For example, while EMPA influenced FGF21 signaling, hypothalamic inflammation, and PDK4 expression in animal studies, corresponding mechanistic evidence in human populations is limited [124, 125]. These pathways play important roles in regulating energy metabolism, insulin sensitivity, and hepatic lipid metabolism; however, their modulation may be less pronounced in humans due to lower therapeutic dosing, greater metabolic heterogeneity, and differences in disease severity. Additionally, hepatic steatosis in human populations is influenced by multiple factors, including genetic predisposition [126], lifestyle variability, and baseline metabolic status [127], which may attenuate the mechanistic effects observed in controlled animal models. These differences likely contribute to the more modest hepatic and metabolic responses observed in clinical trials and emphasize the necessity of human mechanistic studies to confirm whether these molecular pathways are similarly involved in EMPA’s metabolic effects.

### 4.6. Clinical Implications and Future Directions

EMPA, via its insulin‐independent mechanism that facilitates glucosuria and metabolic regulation, has shown beneficial effects on body weight, cardiac and metabolic outcomes, and glucose regulation in patients with T2DM [51, 63, 77, 81]. These properties, along with its favorable safety profile and low intrinsic risk of hypoglycemia, support its potential therapeutic relevance in metabolic disorders beyond diabetes, although its use in nondiabetic populations remains off‐label and requires cautious clinical consideration [11]. Although EMPA is generally well tolerated, careful monitoring of renal function, metabolic parameters, and overall clinical status is recommended, particularly in populations outside approved indications. Across the included RCTs, adverse events were generally mild and infrequently led to treatment discontinuation, with genitourinary infections representing the most consistently reported treatment‐related adverse event. Nevertheless, clinicians should remain vigilant for adverse effects such as genitourinary infections, volume depletion, and rare metabolic complications [128, 129]. Further RCTs are required to elucidate the role of EMPA in nondiabetic individuals with obesity, MASLD, or metabolic syndrome, especially among those exhibiting advanced metabolic dysfunction, where therapeutic benefits may be greater. These trials need to include wider and more heterogeneous populations, extended follow‐up durations, standardized metabolic and hepatic endpoints, and comprehensive safety monitoring to improve generalizability and clinical applicability [75–79, 81]. Additionally, combination strategies targeting complementary metabolic pathways [130], along with incorporation of real‐world evidence [131] and digital monitoring technologies [132], may enhance future trial design by improving patient monitoring, data quality, and representation of real‐world treatment outcomes.

Despite the beneficial effects of EMPA on anthropometric parameters, as well as improvements in glucose regulation and insulin sensitivity in nondiabetic populations, there are some issues that need to be addressed. The search timeframe was deliberately confined to studies published between 2023 and 2024 to prevent the duplication of prior systematic reviews and to deliver an updated synthesis of contemporary clinical and preclinical evidence. Nonetheless, this limitation may have omitted earlier foundational studies, thereby constraining the overall comprehensiveness and generalizability of the findings. Also, although ClinicalTrials.gov was examined to find current or unpublished trials, additional gray literature sources were not routinely checked. This might make publication bias more likely. Moreover, it was impractical to produce aggregated effect estimates from the included studies due to significant diversity in study design, demographics, intervention protocols, dosages, durations, and outcome measurements. As a result, no quantitative meta‐analysis was performed. Moreover, the variability of the study population, the scarcity of RCTs, and methodological constraints necessitate cautious interpretation of the findings. Studies yielding neutral or negative outcomes may be underrepresented, making it hard to eliminate the potential for publication bias. The formal assessment of publication bias using funnel plots or statistical tests proved unfeasible owing to the limited quantity and heterogeneity of eligible clinical trials.

## 5. Conclusion

EMPA generally reduced body weight and improved glycemic control in human studies. However, its effects on lipid profiles and liver enzymes were inconsistent. The limited number of available human trials, with some studies exhibiting methodological concerns, restricts the strength and generalizability of these findings.

Animal studies revealed that EMPA enhanced liver steatosis, glucose control, insulin sensitivity, inflammatory markers, and adiposity, although findings regarding lipid parameters and liver enzymes were variable across studies. Several animal studies also presented methodological limitations, with most being assessed as having an unclear RoB. These findings point out the potential of EMPA as a promising therapeutic option for managing obesity‐related metabolic dysfunctions beyond its glucose‐lowering effects; however, the current evidence remains limited and should be interpreted with caution. Future research should focus on larger, well‐designed, and longer term trials to better delineate the optimal dosing strategies and to further elucidate the underlying mechanisms driving the observed effects in both clinical and preclinical settings.

## Author Contributions

Farima Farsi: conceptualization (equal), literature search and study selection (equal), manuscript drafting (equal); Dorsa Ghorban Sarvi: literature search and study selection (equal), data extraction and quality assessment (equal), manuscript drafting (equal); Mohammadmahdi Abbasi: data extraction and quality assessment (equal), data analysis and interpretation (equal), critical revision of the manuscript (equal); Shirin Hasani‐Ranjbar: conceptualization (equal), data analysis and interpretation (equal), supervision and project administration.

## Funding

The authors did not receive support from any organization for the submitted work.

## Disclosure

All authors approved the final manuscript.

## Ethics Statement

Approval was granted by the Ethics Committee of Tehran University of Medical Sciences (ID: IR.TUMS.EMRI.REC.1403.137).

## Conflicts of Interest

The authors declare no conflicts of interest.

## Supporting Information

Additional supporting information can be found online in the Supporting Information section.

## Supporting information


**Supporting Information** PRISMA_2020_checklist.

## Data Availability

No new data were generated in this study. All data analyzed are available in the published literature included in this systematic review.
